# Immune ontogeny in childhood-onset systemic lupus erythematosus: effect of immune maturity on disease phenotype and therapeutic responses

**DOI:** 10.3389/fimmu.2026.1885364

**Published:** 2026-09-11

**Authors:** Wanfeng Xu, Qingfeng Sun, Meihui Li, Ling Hou

**Affiliations:** 1 Department of Endocrinology, Shengjing Hospital of China Medical University, Shenyang, China; 2 Department of Pediatrics, Shengjing Hospital of China Medical University, Shenyang, China; 3 Class 23, 110^th^ Session, China Medical University, Shenyang, China

**Keywords:** BAFF, childhood-onset SLE, immune ontogeny, interferon, lupus nephritis, precision medicine, stratification

## Abstract

Childhood-onset systemic lupus erythematosus (cSLE) occurs during a period of significant developmental changes in the immune system, when tolerance checkpoints and regulatory networks continue to mature. Compared with adult-onset SLE (aSLE), cSLE more frequently presents with strong type I interferon activity, BAFF-induced B-cell hyperactivation, and increased activity of T follicular helper cells that promote persistent generation of autoantibodies. These changes induce an IFN–BAFF–B-cell–immune complex-complement axis positive feedback loop that can affect developmentally vulnerable organs, thus increasing the risk for early severe lupus nephritis, neuropsychiatric involvement, cytopenias, infection, and drug toxicities. We synthesize evidence for an immune ontogeny-based approach that considers altered molecular pathways, organ development, and responses to different therapeutics, and highlight that a higher genetic burden must be addressed for the effective stratification of patients. Finally, we propose a pragmatic approach to precision care based on developmental stage, immunophenotype, genetics, and long-term safety to enable the earlier onset of steroid-sparing therapy and long-term protection of patients.

## 1 Introduction

Childhood-onset systemic lupus erythematosus (cSLE) is not simply a disease that has an earlier presentation than adult-onset SLE (aSLE). In addition to the earlier onset, cSLE is associated with a higher inflammatory burden, more frequent involvement of major organs, and greater risk of cumulative disease activity, tissue damage, infection, and treatment-related toxicity (1–4). Lupus nephritis, neuropsychiatric involvement, and hematologic abnormalities during periods of growth and organ development are especially severe manifestations in cSLE (1–4).

cSLE occurs during early development, when innate immune sensing, B-cell tolerance, germinal-center selection, T-cell regulation, and hormonal immune modulation are still being calibrated (5–9). Pediatric studies have reported strong type I interferon (IFN) activity and distinct immune-cell signatures in cSLE, indicating that immune maturation may influence the disease phenotype (10–12). These findings provide a rationale for considering IFN activation, B-cell dysregulation, T follicular helper/T follicular regulatory imbalance, and immune complex-complement injury (type III hypersensitivity) in the context of development, although there are different levels of evidence regarding the importance of these pathways.

Recent reviews have summarized advances in the pathogenesis and treatment of cSLE (13, 14). The present review uses immune ontogeny as an organizing framework for understanding why similar disruption of immune pathways in cSLE and aSLE may have different effects on the disease phenotype, organ vulnerability, and therapeutic responses. To avoid overinterpreting the available evidence, we distinguish three levels of support throughout this review: evidence directly from studies of cSLE or pediatric lupus nephritis; evidence from studies of aSLE that may provide insights about cSLE, but that requires cautious interpretation; and hypothesis-generating concepts that require validation in longitudinal pediatric cohorts.

Importantly, most major immune pathways discussed in this review, including type I IFN activation, B-cell dysregulation, and Tfh/Tfr imbalance, are shared by cSLE and aSLE. The distinction in cSLE therefore lies less in the presence of unique immune pathways than in the developmental context in which these pathways operate, their relative magnitude and interaction, the greater contribution of rare disease-driving genetic variants in selected patients, and their consequences for organ vulnerability and lifelong treatment exposure.

**Figure 1:**
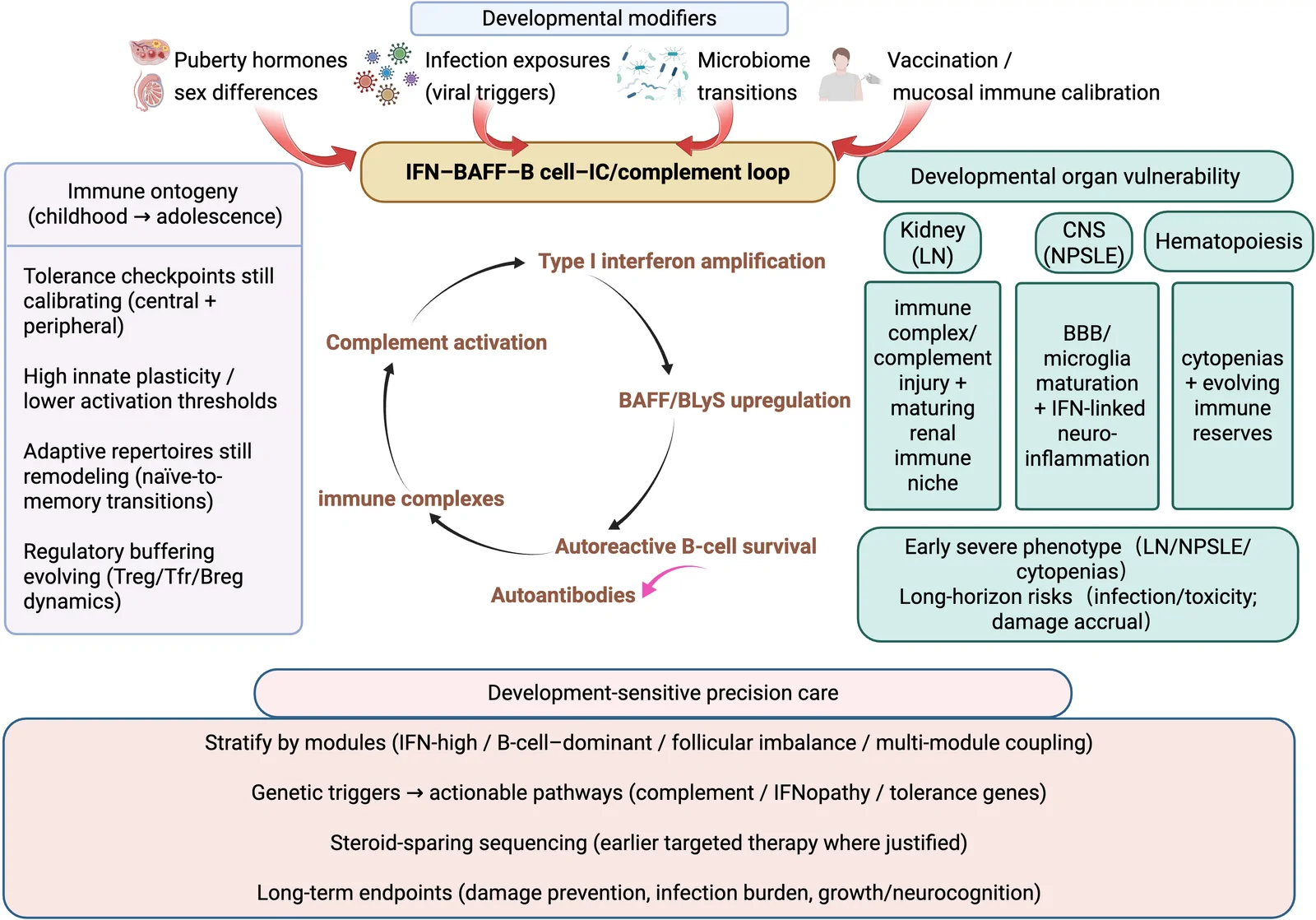
Model of immune ontogeny in cSLE. cSLE onset occurs during immune maturation, when tolerance checkpoints are still undeveloped. In genetically susceptible individuals, developmental and environmental factors, including infections and pubertal hormonal changes, may alter the threshold for immune activation, whereas microbiome-related effects remain hypothesis-generating. These factors may interact with nucleic-acid sensing, type I IFN signaling, B-cell tolerance, and activation of the immune complex-complement axis. Vaccination is considered primarily in relation to immune competence, infection prevention, and treatment safety rather than as an established trigger of cSLE. These immune responses occur in the context of developing organs and may influence clinical phenotype and therapeutic responses (10, 11, 16, 24, 25, 28) (Created in https://BioRender.com).

Within this evidence-stratified framework, we discuss how developmental stage, immune tolerance, immunophenotype, genetic background, organ vulnerability, and therapeutic response may be integrated to guide future risk stratification and trial design (**Figure 1**).

## 2 Immune ontogeny in childhood: a moving target

Understanding cSLE requires a basic understanding of immune ontogeny. During childhood, the increased activity of the innate immune system and the ongoing development of the adaptive immune response and tolerance checkpoints can increase vulnerability to different triggers of autoimmunity and subsequent inflammatory responses.

### 2.1 Development of innate immunity and immunoreactivity

Innate immune responses in children are distinct from those in adults because of age-dependent differences in immune sensing, cytokine production, and regulatory feedback (6, 7). Pediatric studies have reported a prominent type I IFN signature in cSLE, supporting the relevance of nucleic-acid sensing and antiviral response pathways (10, 11). Plasmacytoid dendritic cells (pDCs) can produce large amounts of IFN-α in response to self nucleic acid–immune complexes, a mechanism central to the induction of IFN in SLE (15–17).

More broadly, innate immunity in children is characterized by high responsiveness but limited regulatory stability. Signaling by pattern recognition receptors (PRRs) and the maturation of negative feedback pathways may influence the magnitude and duration of inflammatory responses during childhood (7).

Beyond pDCs, innate recognition and signaling pathways show variable age-related maturation patterns, including developmental differences in Toll-like receptor-mediated cytokine responses (6, 18). These differences may shape responses to self-derived nucleic acids released during infections and tissue injury, although their direct contribution to immune complex-driven inflammation in cSLE remains to be established.

In addition, differences in the development and maturation of antigen-presenting cells (APCs), production of cytokines, and innate immune responses in specific compartments may alter downstream adaptive responses by increasing helper cell and humoral signaling, particularly after exposure to viruses that are encountered during childhood. Together, these findings support the importance of considering developmental stage in cSLE, in which nucleic-acid sensing and IFN-related pathways may contribute to immune amplification (10, 11).

### 2.2 Maturation of adaptive immunity

The peripheral blood of children contains higher proportions of transitional and naive B cells and a decreased proportion of memory B cells (5, 7). Their peripheral tolerance mechanisms, which normally protect against autoimmune diseases, are still maturing, and some autoreactive B cells may escape deletion and become activated in an inflammatory environment. Regulatory B cells also change with age during childhood (4).

Children have a predominance of naive T cells and gradual increases of effector and memory T cells during immune maturation (6, 7). The balance of Tfh cells and T follicular regulatory (Tfr) cells can influence the activity of germinal centers and affinity maturation, and these can alter the selection of autoreactive B cells in cSLE (19).

Importantly, the pediatric B-cell compartment is not only “younger” but is also undergoing development. Transitional B cells, naive B cells, and germinal centers change with age, and the balance of naive B cells, memory B cells, and activated B cells can influence the growth of autoreactive clones after tolerance is breached.

The production of T cells is greater during childhood, but the development of follicular cells can increase in the presence of inflammation. The increased level of Tfh cells and limited follicular regulation in cSLE can promote persistent activity of germinal centers, diversification of the immune response, and the sustained production and evolution of autoantibodies (19–21).

### 2.3 Calibration of immune tolerance

Immune tolerance is maintained through central mechanisms, including clonal deletion and receptor editing, and peripheral mechanisms, including anergy, inhibitory receptor signaling, regulatory lymphocyte activity, and germinal center control. During childhood, the B- and T-cell compartments and their regulatory networks continue to undergo age-related changes (5–7). Inflammatory signaling associated with SLE may disturb this regulatory balance and facilitate the survival or activation of autoreactive clones (10, 11, 16, 19), although the interaction between normal immune maturation and tolerance failure has not been established longitudinally in cSLE.

All of this means that cSLE may be more sensitive to early baseline events that affect pathogenesis. After establishment of autoreactive signaling, the immune system can become locked into self-reinforcing loops (IFN–BAFF–B-cell–immune complexes), a response that may increase the risk of relapse and organ damage (10, 11, 16).

### 2.4 Puberty and developmental modifiers

Puberty is a critical period of immune system development and coincides with an increasing incidence of cSLE. Sex hormones can regulate immune susceptibility by modulating B-cell survival, T-cell differentiation, Tfh-cell activity, and type I IFN signaling (8, 9). Therefore, pubertal status may provide information that is not captured by chronological age alone and should therefore be considered in pediatric cohort studies and clinical trials.

Infection-related immune activation can also modify the clinical manifestations of cSLE. Infections can increase the inflammatory response and may affect nucleic-acid sensing and IFN-related pathways, although the causal relationship between infection and cSLE onset or disease flares remains difficult to establish. From a clinical perspective, serious infections are a major source of morbidity in cSLE and should be considered when evaluating immune reserve, treatment intensity, and long-term outcomes (22, 23).

Vaccination status is clinically relevant in cSLE primarily in relation to infection prevention, immune competence, and the effects of immunosuppressive therapy rather than as an established driver of disease onset. Pediatric studies indicate that vaccine responses may be influenced by the intensity of immunosuppression, reinforcing the importance of reviewing immunization status before and during treatment (24, 25). Vaccination history should therefore be considered as a treatment- and immune-competence variable rather than as a causal developmental modifier of cSLE.

The microbiome may influence mucosal immune education and systemic immune responses, but most evidence linking dysbiosis to lupus has been derived from adult SLE (26, 27). Emerging pediatric evidence provides a potential mechanistic link: a recent cSLE study associated endotoxemia and microbial signals with low-density granulocytes and extracellular-trap formation, suggesting that microbial products may interact with innate immune activation in childhood disease (28). However, direct longitudinal evidence linking microbiome alterations to cSLE onset, flares, or developmental trajectories remains limited. We therefore regard microbiome-related effects as hypothesis-generating rather than as an established determinant of cSLE. Overall, age, pubertal status, and infection history should be considered as developmental or environmental covariates, whereas vaccination status is primarily relevant to immune competence and treatment safety, and microbiome-related effects remain exploratory.

### 2.5 Summary

cSLE occurs in the context of heightened responsiveness of innate immunity, incompletely stabilized adaptive tolerance, and immature immune modulation. This may lower the threshold for autoimmunity and lead to age-dependent differences in immunophenotype and clinical trajectory. Age, pubertal status, and history of infection should therefore be considered as core covariates in cohort studies of cSLE, particularly when longitudinal immune profiling is used to identify actionable biomarkers.

## 3 Breakdown of self-tolerance and immune amplification in cSLE

If immune self-tolerance decreases during childhood, this can manifest as increased autoimmune activity and decreased buffering of autoimmunity. This IFN-centered response consists of amplification of type I IFN signaling, impaired B-cell tolerance, and activation of the immune complex-complement axis. The dysregulation of Tfh cells and the greater genetic burden in cSLE contribute to this pathology (10, 11, 16).

The sections below describe the four major features of the breakdown of self-tolerance and the increased autoimmunity in cSLE:

- Failure of B-cell tolerance and BAFF-driven survival advantages (Section 3.1)
- An imbalance of Tfh and Tfr cells that sustains germinal center reactions (Section 3.2)
- IFN-driven positive feedback loops initiated by the detection of nucleic acids and immune complexes (Section 3.3)
- An increased pediatric genetic burden and the use of patient stratification to make altered pathways clinically actionable (Section 3.4) (10, 11, 16)

This section therefore describes the transition from developmental susceptibility to self-sustaining autoimmunity. In cSLE, altered B-cell tolerance, follicular regulation, nucleic-acid sensing, type I IFN amplification, and genetic susceptibility may interact to stabilize pathogenic immune responses. The following subsections focus on these mechanistic interactions, whereas their immunophenotypic, organ-specific, and therapeutic implications are considered in subsequent sections. This integrated positive-feedback framework is summarized in **Box 1**.

Box 1 **The IFN–BAFF–B-cell–immune complex-complement axis as a positive feedback loop.**

 In cSLE, type I IFN increases antigen presentation and the levels of BAFF, thereby increasing the survival of autoreactive B cells and promoting the differentiation of plasmablasts. Autoreactive antibodies then form immune complexes that deliver nucleic acids to endosomal sensors and engage Fcγ receptors, and this increases signaling by pDCs and myeloid inflammatory cells. Immune complexes also activate the complement system, and this can increase effector injury and impair clearance of the immune complex when there is a deficiency of the classical components, thereby lowering the threshold for sustained autoimmunity. The interactions of these loops can repeatedly “reset” the inflammatory baseline after an infection or tissue injury, leading to organ damage in the developing tissues of children with cSLE (10, 11, 16, 22, 33–39).

### 3.1 Defects in B-cell tolerance checkpoints and escape of autoreactive B cells

B-cell abnormalities are a defining feature of cSLE. During childhood, immune tolerance checkpoints, particularly in the peripheral immune system, are underdeveloped. Inflammation can increase the survival and activation of autoreactive B-cell clones, followed by their differentiation into plasmablasts and plasma cells and the production of autoantibodies (4). Type I IFN signaling can further increase the survival and differentiation of B cells and increase the level of BAFF. This positive feedback loop leads to persistent autoreactivity (3, 29–31).

The failure of pediatric B-cell tolerance in cSLE can be considered as a “checkpoint–inflammation mismatch,” because immune self-tolerance is undeveloped and pro-inflammatory signaling from IFN and BAFF can be disproportionately strong. This mismatch can increase entry of autoreactive clones into germinal centers, where they undergo affinity maturation and diversify into pathogenic autoantibodies.

### 3.2 Dysregulated T-cell regulatory networks

The most serious consequences of T-cell dysfunction in cSLE are increased activity of Tfh cells and decreased activity of Tfr cells, which can increase germinal center activity and the persistent selection of autoreactive B cells (19–21). The dysregulation of Tfh and Tfr cells is particularly relevant for cSLE because the germinal centers affect the quantity, diversity (epitope spreading), and persistence of antibodies. Once a self-sustaining dysregulation is established, immune flares may become less dependent on environmental triggers and more dependent on immune alterations.

Because dysfunctional signaling by Tfh and Tfr cells can affect the risk of relapse, studies of patients with cSLE should perform more frequent sampling during the periods of flares and their transitions to remissions to characterize changes in the balance of Tfh and Tfr cells to identify the specific regulatory defects, rather than solely focusing on downstream changes that are a consequence of increased inflammation (32).

### 3.3 Type I IFN-driven positive feedback loops

The activation of type I IFN signaling is central to the pathogenesis of aSLE and especially of cSLE. Pediatric studies have reported higher levels of IFN and an association of IFN activity with disease activity and involvement of major organs (2). The activation of pDCs by self-nucleic acid–autoimmune complexes initiates IFN-mediated changes in gene expression and increases adaptive autoimmunity (3, 16, 17).

IFN activity can be amplified by repeated exposure to viruses and by immune complex-mediated delivery of nucleic acids to endosomal sensors, leading to recurrent “spikes” that increase the baseline level of inflammation. The innate immune set points of children are immature, so this amplification may be more persistent and may more strongly disrupt downstream signaling in cSLE (15).

### 3.4 Genetic burden and monogenic disease in cSLE

Genetic factors may make a greater contribution to cSLE than to aSLE, particularly in patients with very-early-onset, severe or atypical disease, familial aggregation, or syndromic features. Although most cSLE remains genetically complex, childhood-onset disease provides an important opportunity to identify rare variants with large biological effects (40–42). Monogenic lupus and lupus-like disease can result from defects affecting complement, clearance or sensing of nucleic acids, type I IFN regulation, JAK–STAT signaling, and B- and T-cell tolerance (43–46).

A particularly informative example is TLR7 gain-of-function (GOF) disease. A *de novo* TLR7 p.Tyr264His variant was identified in a child with severe early-onset lupus, and functional studies demonstrated enhanced TLR7 signaling, abnormal survival of BCR-activated B cells, and lupus development in a corresponding mouse model, providing direct evidence that excessive TLR7 signaling can be sufficient to drive lupus (43). More recent reports have expanded the clinical and genetic spectrum of TLR7 GOF-associated disease. In addition, rare variants in UNC93B1, which regulates endosomal TLR7 trafficking and signaling, have been identified in patients with cSLE and linked experimentally to enhanced TLR7/8–IRAK1/4 signaling (45). These discoveries illustrate how genetic studies in cSLE can identify upstream disease drivers that connect nucleic-acid sensing directly with IFN activation and B-cell dysregulation.

Genetic testing should be considered when several key indicators are present: very-early onset of disease, early and severe or rapidly progressive disease, persistent complement abnormalities, the presence of specific signs of primary immunodeficiency, overlapping autoimmune or autoinflammatory manifestations (“lupus-plus”), family aggregation, or other syndromic features. Beyond identifying a molecular diagnosis, genetic evaluation may reveal the dominant dysregulated pathway—for example, complement deficiency, nucleic-acid sensing, IFN activation, or impaired lymphocyte tolerance—and thereby refine risk stratification, family counseling, and, in selected cases, therapeutic reasoning.

**Figure 2:**
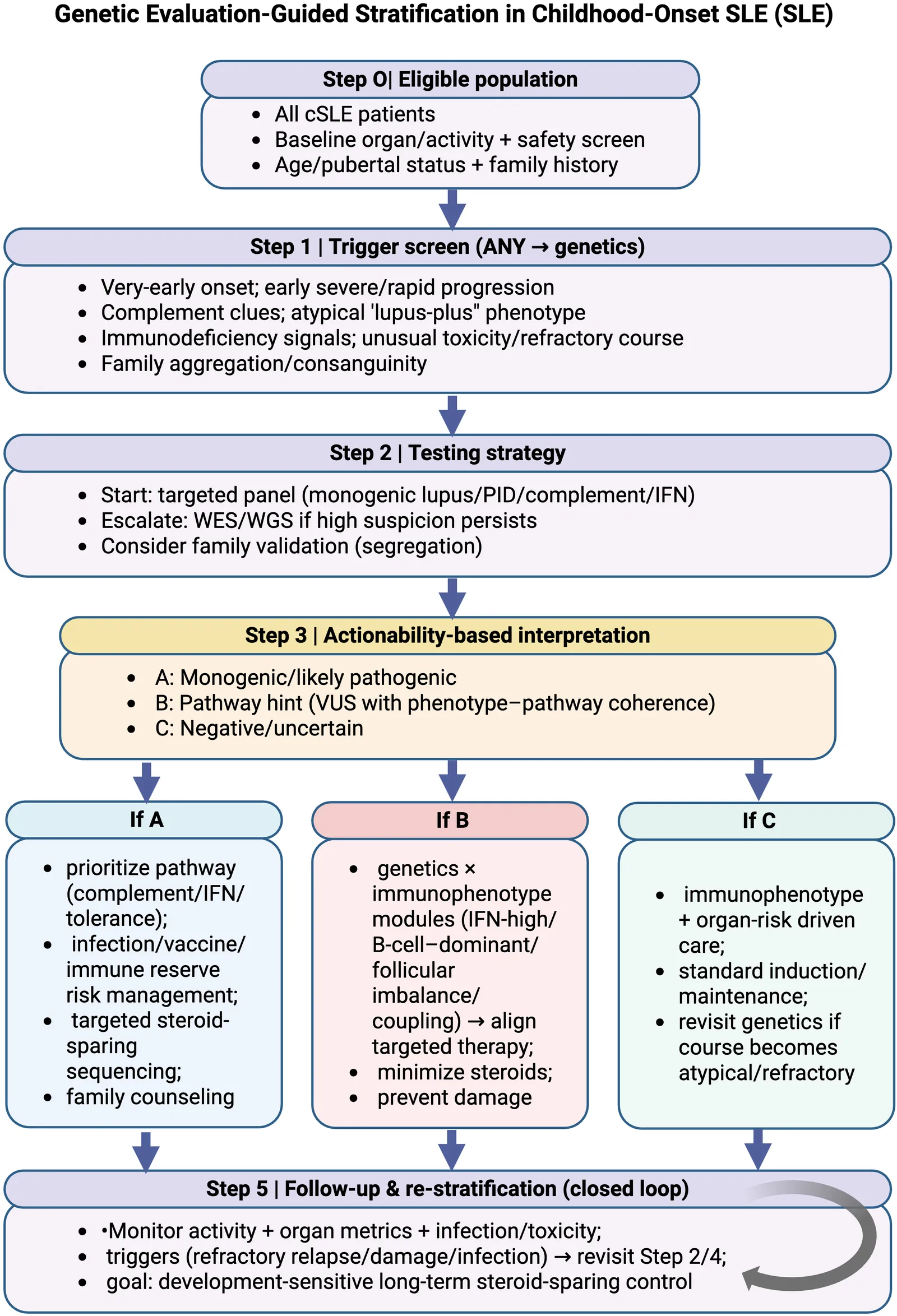
Stratification of cSLE based on genetic testing (created in https://BioRender.com). Workflow for genetic evaluation and stratification of patients with cSLE based on genetics, immunophenotype, and organ risk to identify patients most suitable for steroid-sparing treatment and management of developmental risk.

**Table 1** summarizes the basic indicators for genetic testing, describes their biological basis, and explains their clinical relevance. **Figure 2** presents a workflow that integrates genetic evaluation, immunophenotyping, and assessment of organ risk to support patient stratification and pathway-informed therapy.

**Table 1:** Indicators of monogenic disease or a strong genetic contribution in cSLE.

Indicator	Description	Why it matters
Very-early onset	Onset before age 5–7 years	Higher likelihood of monogenic disease or variants with strong effects; helps explain extreme phenotypes
Early and severe or rapidly progressive disease	Early and severe organ involvement (proliferative nephritis, refractory cytopenias, neuropsychiatric disease, multi-organ involvement)	Suggests strong upstream drivers and pathway dysregulation
Complement alterations	Persistently very low complement or suspected component deficiency; recurrent severe infections with complement abnormalities	Complement deficiency suggests early-onset lupus and severe phenotypes
Atypical “lupus-plus” phenotype	Autoinflammatory-like features, vasculitis-like presentation, disproportionate inflammation with atypical serology	May indicate interferonopathies or autoimmune overlap (beyond simple cSLE)
Immune deficiency signals	Recurrent severe infections, persistent lymphopenia, hypogammaglobulinemia, poor vaccine response	Increased burden of primary immunodeficiency-associated variants; impacts tolerance to immunosuppression
Unusual treatment response or toxicity	Refractory disease requiring extreme immunosuppression, or excessive infection, or toxicity with low-dose therapy	May indicate strong pathway drivers or limited immune reserves; pathway-directed, steroid-sparing treatment may be suitable
Family aggregation	Multiple affected relatives; early-onset autoimmune phenotypes in family members	Higher likelihood of monogenic or recessive inheritance; suggests a need for family counseling
Thrombotic and vascular alterations	Age-inappropriate thrombosis, vasculopathy and microangiopathy after excluding secondary causes	May indicate complement-related or pathway-specific disease
Syndromic features	Coexisting developmental, neurological, endocrine, and skin–skeletal abnormalities	Suggests a syndromic genetic background and the need for broader genomic testing

### 3.5 Summary

In summary, the breakdown of self-tolerance in cSLE can be attributed to interactions of B cells, Tfh/Tfr cells, type I IFNs, and genetic factors. These changes should not be interpreted as isolated mechanisms, because autoreactive B-cell survival, follicular dysregulation, IFN amplification, immune-complex formation, complement activation, and genetic susceptibility may reinforce one another during immune development. These integrated changes support the use of longitudinal immunophenotyping and pathway-informed risk stratification. Emerging multiomics studies of cSLE and single-cell studies of SLE support the mapping of immune heterogeneities into biologically distinct states associated with disease activity and clinical variations (47, 48).

## 4 Immunophenotypes of cSLE

**Figure 3:**
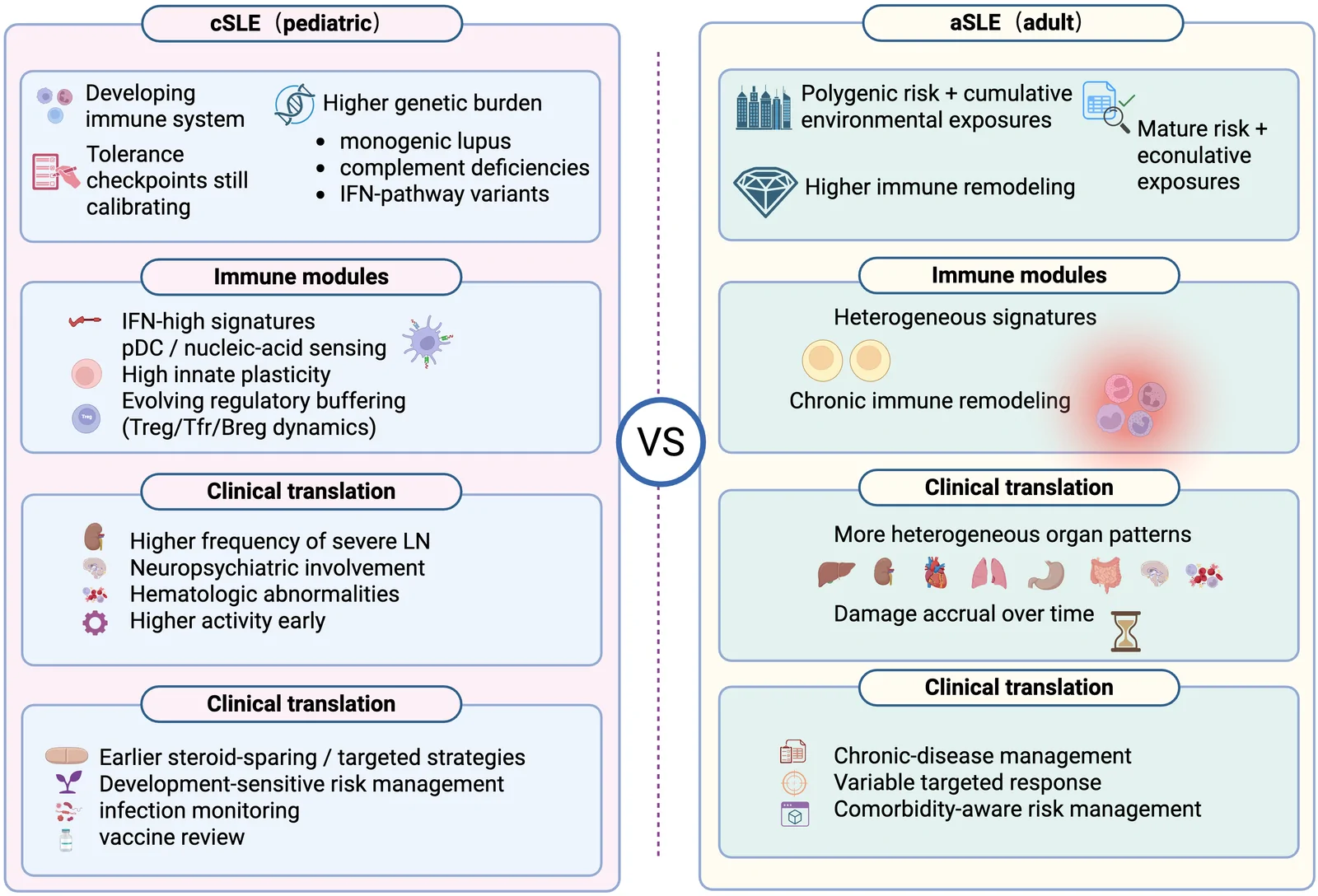
Shared immune pathways in cSLE and aSLE within different developmental contexts. cSLE and aSLE share major immune abnormalities, including type I IFN activation, B-cell dysregulation, and altered follicular helper/regulatory networks. In cSLE, these pathways operate in the context of ongoing immune maturation, a greater contribution of rare disease-driving genetic variants in selected patients, and developing organs. Differences in the relative magnitude and interaction of these pathways may contribute to the earlier and more severe organ involvement observed in some children. This developmental context provides the rationale for evaluating immunophenotype-based stratification specifically in pediatric cohorts (1, 10, 11). (Created in https://BioRender.com).

Patients with cSLE show heterogeneous activation of several immune modules, including type I IFN signaling, B-cell activation, and Tfh/Tfr dysregulation. Importantly, these modules are not unique to cSLE and are also well described in aSLE. Their relevance to childhood disease lies instead in their relative magnitude, combinations, developmental context, and potential interaction with a greater genetic contribution and early organ involvement. **Table 2** compares these features in cSLE and aSLE, and **Figure 3** illustrates how shared lupus pathways may operate differently in the context of immune development. Accordingly, the purpose of modular immunophenotyping in cSLE is not to define pediatric-specific pathways, but to identify biologically meaningful patterns that may improve risk stratification and inform future pediatric studies. Mechanistic pathways are discussed in Section 3; here, we focus on whether these pathways can be operationalized as measurable immune states for pediatric stratification.

**Table 2:** Immune ontogeny, immunophenotype, clinical translation, and therapeutic characteristics of cSLE and aSLE.

Domain	cSLE	aSLE	Clinical and research implications for cSLE
Immune ontogeny	Developing immune system; ongoing calibration of tolerance checkpoints and regulatory networks; higher immune plasticity	Mature immune system; more stable responses and tolerance; chronic inflammatory remodeling over time	Use developmental stage (age and pubertal status) for a biological stratification; prioritize longitudinal designs
Genetic burden	Greater contribution of rare disease-driving variants in selected patients; monogenic lupus includes complement deficiencies, nucleic-acid sensing/TLR pathway defects, and interferonopathies, particularly in very-early-onset, severe, familial, or atypical disease	Predominantly polygenic; greater effects of cumulative environmental exposures; lower monogenic contribution	Consider genetic testing in very-early-onset and severe, familial, atypical cases; or syndromic disease; identification of genetic drivers may reveal actionable biological pathways
Type I IFN axis	More frequent and stronger IFN signatures; often linked to greater disease activity and involvement of major organs	IFN signatures are common but more heterogeneous; influenced by disease duration and prior therapies	Use IFN-high status as a candidate biomarker for stratification; provides a rationale for targeting the IFN pathway
B-cell dysregulation and autoantibodies	Significant B-cell activation, plasmablast expansion, BAFF activity; broader and stronger autoantibody responses	B-cell abnormalities present, but affected by chronicity and treatment; memory B-cell alterations often emphasized	Earlier targeting of B cells as a steroid-sparing treatment in appropriate phenotypes
Regulation of Tfh and Tfr cells	Expansion of Tfh cells; insufficient buffering by Treg cells may increase germinal center responses	T-cell alterations vary; chronic remodeling and memory shifts lead to heterogeneity	Combine T-cell and B-cell modules with IFN status for mechanistic stratification
Organ involvement	Higher frequency of severe lupus nephritis, hematologic abnormalities, and neuropsychiatric involvement; higher activity during early disease	More heterogeneous patterns; damage often accumulates over a longer duration	Identify the most vulnerable organs; emphasize early control and prevention of damage
Infection risk and immune reserves	Ongoing immune maturation; longer lifetime exposure to immunosuppression; higher risk of infection and toxicity in some settings	Influenced by age, comorbidities, and cumulative treatment; risk determinants are multifactorial	Risk management based on developmental stage; need for vaccinations, screening, and monitoring of immune reconstitution
Therapeutic response	Goal: control inflammation while preserving growth and immune development; steroid-sparing strategies prioritized	Goal: control chronic inflammation and prevent long-term damage and comorbidities	Stratification based on developmental stage and immunophenotype; align targeted therapy with dominant modules
Evidence gaps	Fewer RCTs; limited long-term safety and real-world data for biologics	More trial and real-world evidence; complications from response heterogeneity	Need for longitudinal pediatric cohort studies and registries of developmental endpoints

### 4.1 The IFN-high module

Type I IFN activation is a central feature of both cSLE and aSLE and should therefore not be considered a pediatric-specific immunophenotype. However, pediatric studies have reported prominent IFN signatures in cSLE, and some studies suggest that IFN activity may be more intense or sustained than in adult disease (2). The potential cSLE-specific insight therefore lies in the magnitude and developmental consequences of IFN activation rather than in the presence of the pathway itself.

IFN signatures are associated with disease activity and major organ involvement and may therefore contribute to patient stratification. However, clinically useful IFN-high cutoffs must first be standardized across laboratory platforms, and compact gene or protein signatures will be needed for routine pediatric use (2, 49, 50). IFN-high status may eventually identify a subgroup suitable for pathway-directed treatment, but its predictive value and the safety of IFN-directed strategies require direct pediatric validation.

### 4.2 B-cell-driven module

B-cell dysregulation is also shared by cSLE and aSLE and is not intrinsically pediatric-specific. In cSLE, however, autoreactive B-cell activation occurs within a B-cell compartment that is still undergoing age-dependent maturation and tolerance calibration. Pediatric studies have identified plasmablast expansion, BAFF-associated activity, broad autoantibody responses, and immune-complex formation, while recent pediatric lupus nephritis data have further demonstrated remodeling of regulatory B cells and innate lymphoid cells (51). Thus, the relevance of a B-cell-dominant state in cSLE lies in how B-cell activation interacts with developmental tolerance, disease severity, and early organ involvement.

This distinction may also have therapeutic implications. Belimumab has direct randomized pediatric evidence and represents one of the better-supported targeted therapies in cSLE (52). Nevertheless, the biomarkers that identify children most likely to benefit from BAFF inhibition remain uncertain. Adult-derived evidence provides proof of concept: a *post hoc* meta-analysis of the BLISS-52 and BLISS-76 trials suggested a tendency toward greater responses to belimumab among patients with high baseline BLyS protein or type I IFN gene-signature activity, although these exploratory findings require prospective validation and cannot yet be directly extrapolated to cSLE (53). Future pediatric studies should determine whether plasmablast enrichment, BAFF-related signatures, autoantibody trajectories, or combinations of B-cell and IFN markers predict treatment response and long-term organ outcomes.

### 4.3 Tfh/Tfr module

The expansion of Tfh cells and insufficient Tfr-mediated regulation can enhance germinal center reactions, increase the maturation of antibody affinity, and lead to the more sustained production of autoantibodies (19). These abnormalities are not unique to cSLE, but their interpretation in children should consider the ongoing maturation of follicular regulatory networks and their interaction with B-cell activation. Longitudinal pediatric studies are needed to determine whether Tfh/Tfr imbalance identifies a stable disease subtype or instead reflects disease activity, treatment exposure, or developmental stage. Linking this module to clinically meaningful outcomes, including flare frequency, renal response, and cumulative glucocorticoid exposure, will be essential before it can be used for therapeutic stratification (54–56).

### 4.4 Interactions of multiple modules and stratification

The clinical heterogeneity of patients with cSLE may be better explained by the different patterns of interactions among modules, rather than by differences in any single pathway. Accordingly, these modules should be interpreted as quantitative and interacting biological states rather than as cSLE-specific disease categories. High-dimensional profiling and machine-learning approaches have the potential to identify subgroups of patients who are more vulnerable to organ damage and more likely to benefit from specific therapies, and this focus on disease mechanism may enable the improved stratification of patients (6, 47, 50). Single-cell transcriptomics in patients with cSLE can be particularly informative because it can identify the cellular origin of different blood IFN signatures and disease activity-linked states in different immune subsets. This can enable the transition from the use of bulk signatures to modules with actionable definitions (48).

In pediatrics, stratification models should be evaluated by measurements of short-term changes in disease activity indices and by measurements of developmental endpoints, including growth trajectories, the burden of infection, and early markers of damage. These models can be more interpretable, more readily deployed in multicenter settings, and iteratively refined using real-world data (12, 23, 50). Ultimately, modular stratification may be able to identify children for whom earlier use of biological therapies is justified and decrease the use of high-dose steroids, thereby enabling a more normal long-term development. **Table 3** summarizes the effect of specific immune developmental features on phenotype and therapeutic response.

**Table 3:** Effect of immune maturation on disease phenotype and therapeutic response in cSLE.

Developmental feature	Immunologic consequence	Clinical translation	Therapeutic implication
Tolerance checkpoints are undeveloped	Autoreactive clones are more likely to escape peripheral restraint under inflammatory conditions	Broader early serological activity; higher risk of rapid immune amplification	Earlier consideration of steroid-sparing strategies when there is B-cell-dominant biology
High innate plasticity and lower activation thresholds	Stronger or less buffered IFN-I responses to nucleic-acid sensing	IFN-high states may be more clinically consequential	Consider IFN status as a stratification variable, with monitoring of safety when using IFN-directed therapy
Evolving follicular regulation (buffering by Treg cells)	Persistent germinal center activity and epitope spreading	Relapse-prone humoral disease; sustained production of autoantibodies	Align therapy to follicular and B-cell modules; monitor durability of response, rather than activity alone
Developmentally vulnerable organs (kidney, CNS, hematopoietic organs)	Age can alter the tissue phenotype in response to the same immune module	Early severe LN, neuropsychiatric involvement, cytopenias, and infection-limited intensity of therapy	Prioritize organ-protective and developmentally sensitive monitoring; early implementation of strategies to prevent organ damage
Long-term exposure to autoimmunity and treatments	Cumulative toxicity, infection, and developmental disruption have greater consequences	Short-term control is insufficient as a sole endpoint	Appraise growth, neurocognition, infection burden, and prevention of damage when making decisions regarding precision care and trial design

### 4.5 Summary

In summary, the major immunophenotypes described in cSLE largely represent shared SLE pathways rather than pediatric-specific mechanisms. Their potential value in cSLE lies in differences in relative dominance, interaction with immune development and genetic background, and association with early organ involvement. Future pediatric studies should therefore determine whether combinations of IFN, B-cell, and Tfh/Tfr markers provide greater prognostic or therapeutic information than measurements of individual pathways alone.

## 5 Organ vulnerability during development

**Figure 4:**
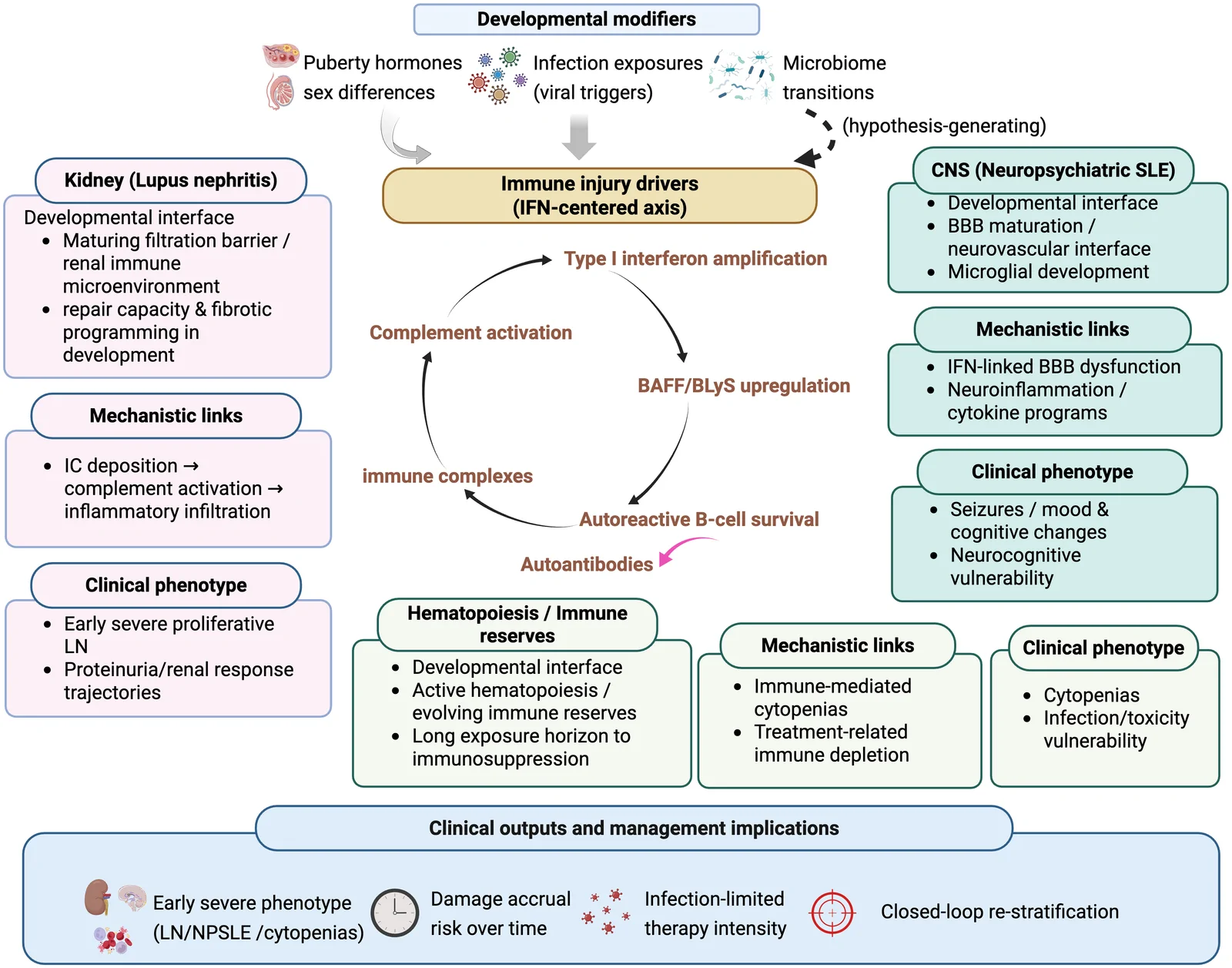
Effect of cSLE on organ development. An IFN-centered immune injury axis (amplification of type I IFN, failure of B-cell tolerance, activation of the immune complex-complement axis) affects the interactions of immature tissues with immune responses, increasing the risk of organ injury. The kidney, CNS, and hematopoietic system—each undergoing maturation—may be particularly susceptible, and their damage may contribute to early and severe lupus nephritis, neuropsychiatric manifestations, cytopenias, and treatment-limiting infections or toxicities. Thus, immune responses to organ immaturity indicate the need for stratified treatments that consider developmental state (23, 58, 59). (Created in https://BioRender.com).

The preceding sections describe the immune mechanisms and measurable immunophenotypes associated with cSLE. This section considers a different question: how these immune abnormalities translate into organ-specific disease in children whose kidneys, central nervous system, hematopoietic system, and other tissues are still developing. **Figure 4** illustrates this developmental organ-vulnerability framework. Rather than proposing organ-specific immune pathways, we focus on how shared lupus mechanisms may have different clinical consequences according to developmental stage and tissue context.

### 5.1 Kidney

Lupus nephritis is among the most common and serious manifestations of cSLE, and children more frequently present with severe proliferative nephritis and high inflammatory activity than adults (5). The immune-complex, complement, and IFN-related mechanisms described in Section 3 provide the mechanistic background for renal injury; the pediatric distinction lies in the early timing and severity of renal involvement and the long lifetime over which renal function must be preserved (1, 2, 11, 33, 54, 55).

In addition, infection-related hospitalization is associated with a higher risk of end-stage renal disease in SLE, particularly in cSLE. This underscores the need to consider control of infections and renal-protective treatments together (34). From a translational standpoint, this emphasizes that “organ severity” is an emergent property of immune dysfunction and organ immaturity. Organ monitoring can therefore be guided by the dominant dysregulated immune modules. For example, intensified renal surveillance should be considered in cSLE patients with an IFN-high/B-cell-coupled state.

This developmental perspective requires early renal surveillance and the timely transition to maintenance strategies that limit the cumulative toxicities of different treatments.

### 5.2 Central nervous system

Neuropsychiatric involvement in patients with cSLE is variable. Systemic dysregulation of the immune system is connected to CNS pathologies by the IFN axis, which can affect interactions of the neurovascular and neuroimmune systems. The modulation of the immune-system maturation and sex-hormone signaling during puberty may also affect the CNS in these patients (8, 9, 36, 57).

It is also necessary to consider neurodevelopmental vulnerability. Inflammation of the CNS during critical periods of development can adversely affect cognitive and psychosocial development, even after resolution of the immediate overt neuropsychiatric events. This strengthens the rationale for use of careful longitudinal assessments and minimizing the cumulative steroid dose and use of potentially neurotoxic agents (cyclophosphamide, methotrexate) when possible.

Studies that identify the links of IFN status with endothelial dysfunction, microglial activation, and specific neuropsychiatric phenotypes remain a priority (37).

### 5.3 Hematopoietic system

Hematologic involvement in cSLE is also common and can arise from autoantibody-mediated damage, defective clearance via complement/Fc receptors, and inflammation-driven suppression of bone marrow. An improvement of immune resilience in patients with long-term cSLE can decrease the risk of infection and increase tolerance of immunosuppressive regimens (23, 38, 58, 59). Serious infection is a major morbidity in these patients, especially those who also have lupus nephritis, and is associated with increased use of corticosteroids (22, 38).

Because hematologic abnormalities and infections can limit the intensity of treatment, approaches that consider the immune reserves and the specific dysfunctional pathways may be helpful. This approach includes a review of vaccination records, screening for latent infections prior to treatment with a biologic, and analysis of the outcomes of patients with infections in hospital registries (22). These measures will be essential because targeted therapies are being introduced at a very young age and will be administered for longer durations.

### 5.4 Disease activity, accrual of damage, and developmental outcomes

Disease activity in cSLE should be assessed by measurements of longitudinal changes, rather than determination of the inflammatory state at a single time, because disease persistence and recurrence may be related to disease flares, organ-specific injuries, responses to glucocorticoids, vulnerability to infections, and overall accrual of damage. The assessment of disease activity should use standardized measures of pediatric disease activity and should target low disease activity by considering the history of flares, organ-specific responses, and exposure to glucocorticoids. Recent cSLE treat-to-target initiatives and the proposed childhood lupus low disease activity state (cLLDAS) are pediatric-specific frameworks that link disease control with prevention of damage and consideration of long-term developmental outcomes (60, 61). Beyond organ injury, cSLE and other pediatric diseases must be understood in the context of growth, puberty, bone metabolism, and neurocognitive development. Chronic inflammation and prolonged exposure to glucocorticoids can affect the growth and long-term outcomes of children, reinforcing the need for a steroid-sparing treatment that prevents organ damage (39, 62, 63).

The developmental outcomes of cSLE patients should be treated as core endpoints rather than “adverse effects.” Growth, the timing of puberty, bone health, metabolic trajectories, and neurocognitive development can all be affected by the sustained inflammation caused by cSLE and by the cumulative exposure to glucocorticoids and cytotoxic agents. This can lead to severe lifelong morbidities in these patients. Consideration of developmental outcomes can also help prioritize which organs should be monitored most closely at disease onset and during flares and the sequence of therapies to be used to prevent irreversible renal and cognitive outcomes and decrease overall cumulative damage. Accordingly, a treat-to-target strategy for cSLE should aim to decrease disease activity, flares, organ-specific responses, steroid exposure, and systemic damage as linked therapeutic goals. Registries of prospective studies should therefore record longitudinal data regarding disease activity, developmental milestones, use of different treatments, and organ outcomes (60, 61).

The long-term pediatric outcomes should include educational attainment and psychosocial function. Even in the absence of overt neuropsychiatric events, chronic disease activity and intensive therapy can disrupt participation and performance at school, and these effects should be recorded using patient-centered outcome measures (63).

Accordingly, “long-term outcomes” in cSLE should be operationalized as a composite of the preservation of renal, CNS, and hematologic systems; prevention of damage; and functional participation in society. These are more suitable targets for treat-to-target and steroid-sparing strategies.

### 5.5 Summary

Recognition of the vulnerability of developing organs provides a mechanistic foundation for the application of development-sensitive therapies that protect long-term outcomes. This approach also explains why some cSLE patients present with early and high-burden organ disease, and why injury of the same immune axis can produce different organ phenotypes in patients at different ages.

## 6 Therapeutic responses in the context of immune development

Patients with cSLE receive treatment during a crucial period of immune maturation and development. Although many therapeutic targets and immunosuppressive agents are shared with aSLE, the major distinction in cSLE lies in the evidence base and the life-course consequences of treatment. Direct pediatric trial data remain limited for many therapies, and treatment recommendations are therefore partly informed by studies of aSLE or mixed-age lupus nephritis populations. Such evidence should be extrapolated cautiously rather than assumed to have identical efficacy and safety in children.

Therapeutic decision-making in cSLE must additionally account for growth, puberty, bone health, fertility, vaccine responses, infection risk, immune maturation, and the substantially longer potential duration of cumulative treatment exposure. These considerations strengthen the rationale for early disease control and glucocorticoid-sparing strategies, but also increase the need for pediatric-specific long-term safety data. Accordingly, the sections below distinguish therapies supported by direct pediatric evidence from those for which efficacy or long-term safety remains largely derived from adult SLE studies.

### 6.1 Conventional immunosuppression

Therapeutic success in cSLE should be determined by measurements of disease activity, organ-specific response, cumulative glucocorticoid exposure, serious infections, and developmental trajectories (growth, bone health, school participation).

Glucocorticoids and conventional immunosuppressants remain an important option for patients with severe cSLE, even though they can impair normal development, especially growth and bone health, and they can increase the risk for infection when given for prolonged periods. Accordingly, pediatric treat-to-target principles and lupus nephritis guidelines emphasize achieving disease control while minimizing cumulative glucocorticoid exposure, with tapering and the introduction of steroid-sparing therapy individualized according to organ involvement, disease severity, and treatment response (60, 64, 65). In clinical practice, conventional immunosuppressive agents are selected primarily according to organ involvement and disease severity, but the immunophenotype should also be considered. Cyclophosphamide remains an important induction treatment for patients with severe proliferative lupus nephritis, neuropsychiatric disease, or life-threatening systemic involvement, circumstances in which rapid and broad immunosuppression is required (64–66). Mycophenolate mofetil is also widely used for induction and maintenance treatment of lupus nephritis and may be preferred when there is a need to decrease renal and humoral inflammation and cumulative cytotoxicity (64–68). Calcineurin inhibitors, including tacrolimus, cyclosporine, and voclosporin, may be considered for patients with prominent proteinuria and podocyte-associated injury, or as part of a multitarget regimen (64–66). These conventional agents are selected primarily according to organ involvement, disease severity, renal pathology, previous treatment response, toxicity, infection risk, fertility considerations, and the anticipated duration of therapy. Although immunophenotyping may eventually refine treatment selection, there is currently insufficient pediatric evidence to use IFN-high, B-cell dominant, or other immune modules as validated biomarkers for choosing among cyclophosphamide, mycophenolate mofetil, and calcineurin inhibitors. Thus, immunophenotype-guided selection of conventional immunosuppression should presently be regarded as a hypothesis for prospective pediatric studies rather than an established treatment strategy.

An induction regimen is therefore useful for patients with cSLE, but the toxicity profiles and developmental consequences of glucocorticoid therapy in children require the use of the “lowest effective exposure.” This includes proactive steroid tapering, early transition to maintenance therapy, and the structured monitoring of growth, bone health, metabolic parameters, and infections. When available, a treat-to-target approach and standardized measurements of disease activity and organ damage can inform the intensity of induction therapy with longitudinal goals. This approach can decrease variations in care and facilitate comparisons of treatment efficacy in different institutions (64–66).

### 6.2 Targeting the B-cell axis

Among targeted therapies, belimumab has a comparatively stronger pediatric evidence base. A randomized placebo-controlled trial demonstrated efficacy and acceptable safety in children with cSLE (52, 69–71), and pediatric real-world data provide additional supportive evidence (52, 69–71). In contrast, much of the longer-term evidence regarding sustained safety and organ damage accrual has been generated in adult SLE cohorts (52, 69–71) and should be extrapolated with caution to children.

BAFF inhibition is biologically relevant to the B-cell abnormalities described in cSLE, but biomarker-guided selection remains insufficiently validated. Earlier use of belimumab in selected children may support glucocorticoid-sparing treatment goals; however, whether plasmablast enrichment, BAFF-related signatures, autoantibody trajectories, or combined B-cell/IFN profiles predict treatment response remains uncertain. Longitudinal pediatric studies are therefore needed to determine which patients benefit most and whether earlier B-cell-directed treatment improves long-term renal, developmental, and damage-related outcomes.

The implementation of this therapy must consider its timing relative to induction therapy, measure biomarkers of B-cell-dominant biology (e.g., plasmablast signals, BAFF level), and integrate B-cell targeting with mycophenolate maintenance therapy for nephritis (67, 68, 72, 73). To simplify the selection of different therapies, decision tools can be structured as “module-first” (IFN-high vs. B-cell-dominant), with consideration of interactions with developmental and genetic factors. This framework provides a clinically intuitive basis for the selection of treatments and facilitates the identification of biologically homogeneous patient subsets that can be used for clinical trials (10, 11, 16).

### 6.3 Targeting the IFN pathway

Anifrolumab provides a clear example of the limitations of adult-to-pediatric extrapolation. Evidence supporting type I IFN receptor blockade is currently derived predominantly from trials in aSLE (74), and comparable efficacy and safety cannot yet be assumed in cSLE. Pediatric evaluation should specifically address infection risk, vaccine responses, immune memory, and long-term developmental outcomes because type I IFNs have important roles in antiviral defense and immune programming during childhood. Thus, an IFN-high state represents a biologically plausible enrichment strategy for future pediatric trials, but it is not yet a validated biomarker for treatment selection in routine cSLE care. As pediatric data increase, trial designs that incorporate IFN-high enrichment, organ-specific endpoints (e.g., renal response), and steroid-sparing outcomes will be essential to define the role of IFN blockade in cSLE (64, 65).

### 6.4 Dual stratification based on immune ontogeny and immunophenotype

A precision strategy that combines developmental context (age, pubertal stage, immune reserves, infection history, vaccination status) with immunophenotype and the interactions of different modules (IFN-high, B-cell-driven, Tfh/Tfr imbalance, and multiple modules) may be especially effective. Analysis of the genetic burden can help to determine which upstream pathways should be targeted and decrease risk (12, 40–42). This approach can facilitate clinical decision-making and, when applied to clinical trials, can increase the statistical power of studies (10, 11, 16). At present, however, this dual-stratification approach should be regarded as a testable research framework rather than a validated treatment algorithm for routine pediatric care.

Over time, this approach can be used to develop interpretable decision-support tools that recommend “next-best” steroid-sparing options, with “flagging” of patients who need more intensive surveillance of infection or genetic evaluation (12, 23, 50).

### 6.5 Summary

In cSLE, therapeutic decisions should distinguish established pediatric evidence from adult-derived evidence and balance rapid disease control against cumulative treatment toxicity and developmental safety. Glucocorticoid-sparing strategies are particularly important because treatment begins early in life, but immunophenotype-guided selection of therapy remains insufficiently validated. Future pediatric studies should therefore evaluate not only short-term disease and organ responses but also cumulative glucocorticoid exposure, serious infection, growth, immune reconstitution, and other long-term developmental outcomes.

## 7 Future perspectives

### 7.1 Longitudinal cohort studies

Longitudinal studies that consider the stage of immune system development are indispensable for making causal inferences regarding cSLE. Without these studies, the immunophenotype cannot be linked to the occurrence of disease flares, organ damage, and the long-term accumulation of injury. A key priority is to establish longitudinal pediatric cohorts spanning early disease (near onset) and tracking immunophenotypic changes during development, including the transition through puberty. Such cohort studies should perform repeated measurements of immune profiles using cellular, transcriptional, and humoral measurements and should use standardized clinical endpoints, such as disease activity indices, organ injury, accrual of overall damage, and growth and developmental outcomes. Study designs that focus on immune development will help clarify temporal relationships among IFN activation, B-cell dynamics, and organ injury and help to determine when and for whom early targeted interventions are most suitable.

### 7.2 Translational biomarkers

The principal challenge is no longer simply to identify additional immune abnormalities in cSLE, but to translate the immune states described in Section 4 into reproducible biomarkers that can be used across centers. Candidate biomarkers should be development-sensitive, analytically standardized, and able to predict clinically meaningful outcomes such as disease activity, organ damage, treatment response, or toxicity. High-dimensional immunophenotyping, transcriptomics, proteomics, and single-cell approaches may identify candidate signatures, but clinical translation will require minimal and reproducible panels that can be prospectively validated in pediatric cohorts. A practical near-term goal is therefore to test whether composite biomarker panels provide greater prognostic or predictive value than individual immune markers.

### 7.3 Long-term safety and real-world evidence

Duration of treatment and developmental state are critical biological variables in cSLE and other pediatric diseases. The need for long-term treatments means that there is a need to focus on drug safety and follow-up examinations that determine immune maturation, risk of infection, and cumulative toxicity. The long-term follow-ups must consider organ outcomes, as well as the risk of infection, immune reconstitution, response to vaccinations, effects of treatments on the endocrine system and growth, bone health, neurocognitive development, and psychosocial functioning. If biologics and pathway-directed therapies are introduced earlier, real-world registries can be designed to quantify the trajectories of these patients, the durability of disease control, and developmental endpoints. Targeting the IFN pathway in cSLE should be accompanied by structured monitoring for infectious complications and longitudinal immune maturation (10, 11, 16).

### 7.4 Bridging research and clinical practice

Precision care can be scaled into larger populations only if immune stratification becomes clinically interpretable. This can be achieved using a simplified and validated decision framework that integrates different modules, developmental states, genetics, and risk of organ injury. Studies that employ machine learning may be able to identify subgroups of patients based on immunophenotype, but clinical adoption of these results depends on transparent machine-learning features, standardized assays, and evidence that stratification affects outcome. Clinical decision frameworks that consider the developmental stage of patients with cSLE should therefore seek to integrate four key variables: (i) immune modules (IFN, B-cell, follicular helper/regulatory circuits), (ii) developmental context (age, pubertal stage, immune reserves), (iii) genetic pathway analysis (monogenic disease, complement/IFN-related variants), and (iv) the longitudinal risk of organ injury. A trigger-based genetic evaluation (**Table 1**) and workflow-based stratification (**Figure 2**) show how mechanistic insights can be implemented in clinical practice. Future studies should validate these tools prospectively so that they can be used to care for different patients (10, 11, 16).

## 8 Conclusion

cSLE can be considered an autoimmune syndrome arising within a developing immune system, in which developmental stage influences immune tolerance, immunophenotype, organ vulnerability, and therapeutic consequences. Most major immune pathways are shared with aSLE; the distinction in cSLE lies in its developmental context, the greater contribution of rare disease-driving genetic variants in selected patients, the early burden of major organ disease, and the lifelong consequences of treatment. This perspective provides a more precise basis for understanding the heterogeneity of childhood lupus than simply regarding cSLE as an earlier presentation of adult disease.

cSLE is best understood as a failure of immune ontogeny, and not by the simplistic view that it is merely an earlier presentation of aSLE. A patient’s developmental stage should be treated as a biological variable that can affect upstream triggers, buffering of immune responses, susceptibility of different organs, and balance of therapeutic response and toxicity in different treatments. This perspective suggests a need to change the goal of treatment from simple inflammation suppression toward development-sensitive remodeling of the immune system. This can potentially be achieved by steroid-sparing strategies in patients with early disease, alignment of targeted interventions with the predominant immunophenotype, and long-term protection of growth, development, immune maturation, and overall function.

The integration of data about immune ontogeny, immunophenotype, organ vulnerability, and signaling of different genetic pathways provides a coherent roadmap for precision treatment of cSLE. These treatments should aim to control disease activity and prevent the early damage of organs, without creating an economic burden for patients. The most impactful therapeutic advances will likely come from trials that consider the different immunophenotypes and genetic pathways, begin with steroid-sparing therapies, and measure outcomes during development of the immune system, while preserving organ function and preventing adverse effects.

From this perspective, therapeutic success in cSLE should be defined not simply by the short-term suppression of inflammation. Instead, it is important to achieve a sustained protection of organs, to minimize the cumulative exposure to glucocorticoids, and to preserve immune competence and developmental trajectories (75). These benchmarks are particularly important in children, because disease activity and treatment-related toxicities can affect growth, education, psychosocial function, and lifelong organ outcomes.
